# Gaming‑Based Community Intervention for Loneliness in Adult Gamers: Longitudinal Observational Study

**DOI:** 10.2196/82428

**Published:** 2026-02-10

**Authors:** Christopher Neu, Thomas D Hull, Matteo Malgaroli

**Affiliations:** 1 Hero Journey Club San Francisco, CA United States; 2 New York University Grossman School of Medicine Department of Psychiatry New York, NY United States

**Keywords:** gaming interventions, video games, adult gamers, discord, loneliness

## Abstract

**Background:**

Loneliness is a form of psychological distress associated with increased risk of depression, anxiety, and adverse health outcomes across the life span. This study evaluates an online gaming‑based community intervention that combines professionally facilitated groups, commercial video games, and skills‑focused workshops for adults who play video games.

**Objective:**

This study aimed to examine the feasibility of this health‑supporting gaming community and to characterize 30‑ and 60‑day changes in depression, anxiety, psychological well‑being, and psychological flexibility, as well as heterogeneous trajectories of depressive symptoms.

**Methods:**

In a longitudinal observational study, adults in the United States self‑enrolled in a gaming therapeutics community hosted on Discord. Participants completed baseline, 30‑day, and 60‑day assessments including the Patient Health Questionnaire‑9 (PHQ‑9), Generalized Anxiety Disorder‑7 Scale, World Health Organization-5 Well‑Being Index, and Psychological Flexibility Scale (Psy‑Flex). Of 438 participants with 30‑day data, 403 met inclusion criteria for longitudinal analyses and 157 (35.6%) completed the 60‑day survey. Within‑person change scores and standardized mean differences were calculated, and latent growth mixture modeling was used to identify depressive‑symptom trajectories and baseline predictors of nonresponse versus improvement.

**Results:**

At baseline, mean PHQ‑9 score was 13.37 (SD 6.04), decreasing to 10.27 (SD 5.80) at 60 days (Cohen *d*=0.52). Mean Generalized Anxiety Disorder‑7 scores decreased from 11.23 (SD 5.24) to 8.25 (SD 4.22) (Cohen *d*=0.60). Psy‑Flex scores increased from 11.51 (SD 4.30) to 12.55 (SD 4.37; Cohen *d*=0.24), and World Health Organization‑5 Well‑Being Index scores increased from 7.86 (SD 3.82) to 8.08 (SD 4.44; Cohen *d*=0.24). Latent growth mixture modeling identified 3 depressive‑symptom trajectories: a low group (229/438, 52.3%; baseline PHQ‑9 mean 8.80 (SD 3.67); 60‑day mean 7.64, SD 3.87), a chronic group (118/438, 26.9%; baseline mean 18.30 (SD 4.03); 60‑day mean 16.78, SD 4.10), and an improvers group (91/438, 20.8%; baseline mean 18.52, SD 3.13; 60‑day mean 7.42, SD 4.27). In logistic regression among participants with moderate‑to‑severe baseline depression, a gender identity other than woman was associated with lower odds of belonging to the Improvers versus Chronic group (odds ratio 0.42, 95% CI 0.19-0.94). Post hoc analyses indicated lower odds of improvement for nonbinary participants compared with women (odds ratio 0.25, 95% CI 0.10-0.59). No other baseline characteristics significantly distinguished chronic versus improving trajectories.

**Conclusions:**

A professionally moderated, gaming‑based community intervention was feasible to deliver, engaged a diverse sample of adult gamers, and was associated with medium‑sized reductions in depressive and anxiety symptoms and small improvements in well‑being and psychological flexibility over 60 days. A subgroup moved from moderate‑to‑severe symptoms to subthreshold depressive symptoms. These findings support further controlled evaluation of health‑supporting gaming communities as a scalable support and potential preventive context for adult gamers experiencing distress.

## Introduction

Loneliness is a form of psychological distress that has been linked with increased risk of several serious medical conditions like diabetes, stroke, heart disease, dementia, depression, anxiety, suicide, and self-harm [1-6]. By the age of 25 years, almost 80% of individuals will have experienced loneliness at some point in their life, along with the associated health risks like depression, anxiety, suicidal ideation and self-harm, and will likely experience it again as they age [4,7,8]. Due to the increased risk of several severe diseases and mental health risks, loneliness is being identified by large health organizations, such as the Centers for Disease Control and Prevention, World Health Organization, and American Psychological Association, as a critical point of intervention for increasing health and wellness of individuals [1,7,9,10].

Importantly, while increasing prosocial behavior and interaction with a community is commonly proposed, increased social contact is insufficient to decrease loneliness on its own [11]. Conditions such as social anxiety, social skills, self-esteem, and personal distress can all decrease the likelihood that connection with community improves experiences of loneliness [12,13]. This is especially true for marginalized populations, including individuals that are neurodivergent, LGBTQIA+ (lesbian, gay, bisexual, transgender, queer or questioning, intersex, asexual, and others), or Black, Indigenous, and People of Color (BIPOC) that find it difficult to navigate social situations where connection and welcomeness may be ambiguous [14].

Online communal servers and chat services often provide a sense of community and virtual relationships [15], but these communities are often not designed specifically for connection or mental health. Online video game communities can be hostile or “toxic,” which does not promote meaningful connection that improves mental health [16-18]. Additionally, gaming has ambivalent attention within the mental health community, given concerns about addiction and social withdrawal [19-21]. Due to this, many have steered away from the potential of using video games, when appropriately designed and supported, to build social connections, community and to combat loneliness. In contrast to these largely unstructured and sometimes “toxic” gaming spaces, the present intervention was intentionally designed as a health‑supporting community. It uses explicit community guidelines, structured channels and events, active professional moderation, and therapist‑facilitated groups to promote supportive interactions and reduce exposure to harassment, discrimination, and other harmful content.

Research on the mental health of video game players reveals that the gaming community is often distressed, with low physical, social, and mental health reported [22-24]. However, a common belief is that problematic video game use is a cause or catalyst to lower well-being, research suggests that problematic use is a coping strategy for distress rather than the cause [12,19,25,26]. Recent research further highlighted the moderating role that meaningful relationships, especially parent-child relationships have on problematic video game usage and aggressive behavior linked to violent video games [27-30]. This perspective suggests using a systemic lens to evaluate the promises and perils of video games for supporting well-being and community cohesion. Furthermore, video games have been associated with a number of cognitive and mental health benefits, both in the absence of and in addition to traditional treatment options [31-38].

The interpersonal structure of online communities and the documented benefits of gaming suggest an opportunity to reimagine gaming platforms as accessible digital settings for challenging loneliness and promoting well-being. However, given the complex relationship between gaming, online toxicity, and mental health, there is a critical gap in understanding how gaming environments might serve as therapeutic environments. To fill this gap, in this study we investigate whether a communal gaming environment intentionally designed to promote mental health is a feasible and acceptable intervention for improving well-being at 30 days and 60 days. This approach was designed to leverage the unique characteristics of gaming communities and of their online environment to expand individual capabilities combined with group and community interventions that offer new, supportive opportunities to build relationships are essential for breaking self-sustaining feedback cycles that contribute to ill health.

## Methods

### Setting and Participants

This study is a proof-of-concept feasibility study of a health-supporting online community using a longitudinal observational design. Participants consisted of self-referred users of an online community. Recruitment occurred via targeted social media posts on platforms frequented by gamers (eg, Discord [Discord Inc.], X [X Corp], and Reddit [Advance Publications]), outreach to existing gaming communities, and word‑of‑mouth referrals from current participants. The structure and reporting of this paper follow the guidelines of the STROBE (Strengthening the Reporting of Observational Studies in Epidemiology) Statement for observational research (Multimedia Appendix 1). Sample selection was nonrandom with primarily purposive and snowball sampling methods. Sample size was based on the feasibility of the program’s first phase rather than an a priori calculation. Participants of this group model were included if they were older than 18 years and had access to discord. Participants were excluded from participation if, at sign up, the participant was evaluated to have a condition unsuitable for a subclinical service, such as active suicidality or homicidality, active psychotic symptoms, or ongoing substance use disorders. The final sample consisted of 438 participants.

### Program Procedures of Health-Supporting Communities

Systems theory is a useful framework for understanding how patterns in couples, families, groups, and in this case, broader communities shape individual thoughts, emotions, and behavior [39-42]. Rather than focusing only on one‑to‑one treatment, systems theory highlights opportunities to relieve distress by changing the social contexts people inhabit, in ways that complement but do not replace individual care [40,43-45]. Guided by this systems‑theory perspective, the gaming community was intentionally structured to address both internal systems of the self (eg, beliefs, emotion‑regulation, and interpersonal skills) and external social systems (eg, patterns of interaction in the Discord server and journey groups). In the following sections we give an overview of the theoretical basis, programs, and models we use that were developed with both external and internal system perspectives in mind. The online nature of this program does present potential risks including emotional discomfort or distress due to the discussions in group meetings on discord, the inherent risk of negative interactions from peers in an online community and group setting, as well as possible disclosure of personal issues by peers in groups or communities in a way the individual does not desire. For all of these, efforts were made to reduce risks through online moderation, discussion and reminders of confidentiality in every group meeting, and journey guides (or group leaders) that are trained mental health professionals.

### Building Health-Supporting Communities

#### Peer Relationship Building: Discord Communities

Discord is a platform for social networks, primarily used by gamers, to communicate and build community around a specific topic or game. We organized several “servers” or subcommunities around shared gaming interests. Each server is overseen by moderators and staff, and offers text and voice channels, read-only resources, event spaces, and community rules to encourage and preserve supportive interactions. These online communities are designed to aid in meaningful and health-promoting connections between members [46]. Careful, active moderation of this community facilitates the intended purpose of reinforcing social skills and preserving safety of all parties [13,47]. While the interactions are inherently unstructured, each participant reads and acknowledges the rules of the discord server which are moderated and enforced. These moderation rules are listed in Appendix 4.

#### Journey Sessions

In addition to Discord communities, weekly group sessions are facilitated by group leaders with counseling or clinical training. A group setting has been selected because group settings have long been found to be effective for providing social support to individuals experiencing distress [48-51] and can be a suitable setting for teaching self-care, social and emotion regulation skills, and offers a practical setting for practicing these skills [48].

This intervention is designed to position peer support as a complement to, rather than a replacement for, trained clinical guidance. Peer-based group interactions, facilitated through cooperative video game play, offer unique benefits, including normalization of social struggles, opportunities for mutual identification, and repeated exposure to low-stakes social engagement that can reduce loneliness through increased belonging and shared purpose [52,53]. However, peer communities alone may be limited in their ability to systematically target maladaptive social cognitions, address avoidance patterns, or scaffold skill acquisition in an appropriate manner. Trained facilitators and structured psychoeducational components can address these gaps by providing evidence-based frameworks (eg, behavioral activation, social skills training, and cognitive models of loneliness), providing psychological safety, and guiding reflection that links in-game social experiences to real-world interpersonal functioning [54,55]. Journey guides (group facilitators) shape norms, reinforce adaptive behaviors, and translate emergent peer interactions into durable skills and insights. In this structure, peers provide authenticity, immediacy, and belonging, while journey guides aid groups in adhering to mechanisms of change and mitigating known risks of unguided peer support [56]. Journey session structure is outlined in Appendix 2.

Commercial video game play (ie, *Minecraft, Animal crossing*, etc) is reported to accelerate group cohesion and bonding through shared interests [46], and provides opportunities to explore individual and group dynamics, as well as practice learned skills [57]. Socio-emotional skills are drawn from well-validated interventions such as Dialectical Behavior Therapy [58,59] and Acceptance and Commitment Therapy [60]. Group members are connected in a text channel on Discord that is accessible only by that particular group to foster connection and support throughout the week in between sessions. Group meetings provide a context of support [61,62], accountability, and normalization of challenges and setbacks as skills are learned and practiced [63-65].

#### Working With Imminent Risk

Protocols were designed and followed for imminent risk within this subclinical, online support program. Guides were not acting as treating clinicians and contact with journeyers occurred in an online environment. These protocols included the following steps. The first step is a crisis-resource channel in the discord. Journeyers were advised frequently throughout participation about the resources within this page. Participants were also instructed how to use the crisis-resource page which included numbers to centers and hotlines. The protocol for addressing imminent threat is included in Appendix 3.

#### Gaming Therapeutics

Gaming therapeutics use readily available commercial games to further practice skills through preconstructed situations akin to an interactive worksheet set in the game world. They are especially helpful for emotion regulation, executive functions, problem solving, decision-making, and social collaboration [15,48,57,66]. Games like *Minecraft, Roblox, Skyrim,* and *D&D* have a developer mode that allows designers to create scenarios for specific skill building exercises.

### Weekly Session Facilitators

We had a sample of 36 facilitators for these groups. Of these, 11 (31%) reported social work training, 9 (25%) reported art therapy training, 1 (3%) reported a Doctor of Psychology, 8 (22%) reported marriage and family therapy training, 7 (19%) reported mental health clinician training. The average age of these facilitators was 32 (SD 4.56). They collectively represent 25 different geographic states in the United States with the most common states being New York, Florida, and Texas. Regarding gender, 28 (78%) facilitators identified as women, 4 (11%) as men, and 4 (11%) non-cis-gendered. All weekly journey groups were led by “journey guides,” who were licensed or prelicensed mental health professionals (eg, social work training, marriage and family therapy training, mental health clinician training, art therapy, and Doctor of Psychology) contracted by the program. Before leading groups, guides completed a structured onboarding that included asynchronous training modules on the program model, Discord community guidelines, group structure, and crisis‑escalation procedures, followed by live role‑play sessions and observation of at least 1 full group cycle. Guides were provided with written session outlines (check‑in prompts, group agreements, theme‑setting questions, and closing prompts) to support consistent delivery while allowing flexibility to respond to group needs, and they used standardized protocols for moderating chat, handling rule violations, and escalating risk concerns. Throughout the program, guides attended regularly scheduled group supervision meetings (biweekly) with the Director of Clinical Operations and could request ad‑hoc consultation for complex cases, including any incidents involving crisis‑resource referrals or duty‑to‑warn decisions.

### Measures

#### Demographic Questions

Participants completed demographic questions including gender identity, race and ethnicity, and self-reported neurodivergent status. These questions were asked during onboarding.

#### Patient Health Questionnaire‑9

We administered the Patient Health Questionnaire‑9 (PHQ-9) at entrance to the program and again at 30 days and 60 days via survey. The PHQ-9 is a self-report screener of depression symptoms and severity that has been evaluated as a reliable and valid measure for several populations. There are 9 questions with answers ranging from 0=Not at all to 3=Nearly every day. Symptoms measured include: “Little interest or pleasure in doing things” or “Trouble concentrating on things, such as reading the newspaper or watching television.” Due to its relatively short length and sensitivity to change over time [67], it is an ideal measure for gauging distress over time.

#### Generalized Anxiety Disorder‑7 Scale

We also used the Generalized Anxiety Disorder-7 Scale (GAD-7) to measure anxiety symptoms and severity [68]. Like the PHQ-9, this was administered via survey at the start of the program, and then again at 30 and 60 days. This was a 7 item questionnaire that measured symptoms over the last 2 weeks. Each item could be answered from 0=Not at all to 3=Nearly every day. Items include: “Trouble Relaxing” and “Feeling afraid, as if something awful might happen.” The GAD-7 was also selected for its length, reliability, validity, and sensitivity to change [69].

#### World Health Organization-5 Well‑Being Index

The World Health Organization‑5 Well‑Being Index (WHO-5) is a brief, 5‑item self‑report measure of subjective psychological well‑being developed by the World Health Organization [70]. Items assess positive mood, vitality, and general interest over the past 2 weeks (eg, “I have felt cheerful and in good spirits”), with responses scored from 0 (“at no time”) to 5 (“all of the time”). Item scores are summed to yield a total score from 0 to 25, which is then multiplied by 4 to produce a 0-100 index, where higher scores indicate better well‑being. The WHO‑5 is widely used as a generic measure of mental well‑being and has demonstrated good reliability and construct validity across clinical and non‑clinical populations, including digital mental health and community samples. In this study, the WHO‑5 was administered at baseline, 30 days, and 60 days to index changes in positive well‑being alongside symptom measures.

#### Psychological Flexibility Scale Inventory

The Psychological Flexibility Scale (Psy-Flex) was also administered at baseline, 30 days and 60 days [71]. This is a 6 question assessment of psychological flexibility. Statements are measured over the last 2 weeks and include items such as: “Even if I am somewhere else with my thoughts, I can focus on what's going on in important moments.” and “I can look at hindering thoughts from a distance without letting them control me.” Scores for each question range from 1 to 5 and total scores are summed. This measure was chosen for its short length as well as its connection to the psychological flexibility literature, in which greater psychological flexibility is associated with reduced symptoms and better functioning across a range of mental health concerns. In this study, psychological flexibility was examined as a candidate process through which participation in skills‑focused groups and community activities might support improvements in depression, anxiety, and well‑being [71].

### Data Analysis

Data were pulled from the program in January of 2024. Demographic data were gathered for participants who responded to an invitation to complete measures about their experience in the program. Only those who had at least 2 time points were included in the analysis. Participants were then separated into 2 groups based on their baseline scores for the GAD-7 or PHQ-9.

To model changes in depressive symptoms following enrollment, we conducted Latent Growth Mixture Modeling (LGMM) to identify distinct subgroups characterized by different PHQ-9 symptom trajectories over 60 days in the subclinical support program. We focused trajectory modeling on PHQ-9 scores given the higher comorbidity between loneliness and depression [1] as well as sample size considerations. We used an intent-to-treat approach for the LGMM by including all participants and handling missingness through full information maximum likelihood. We modeled nested unconditional LGMMs with increasing numbers of trajectories (1-4). All models included random effects for the intercept and slope parameters and a fixed quadratic growth factor to capture potential nonlinear change patterns in depressive symptoms over time. The best fitting model was determined using a combination of fit indices and theoretical considerations [72]. Examined criteria included Bayesian information criterion, sample-size adjusted Bayesian information criterion, Akaike information criterion, relative entropy, and statistical significance of the Lo-Mendell-Rubin likelihood ratio test and the bootstrap likelihood ratio test. Additionally, we considered parsimony, class interpretability, and clinical relevance when selecting the final model [72]. After determining the best fitting LGMM solution, we conducted logistic regression analyses to identify baseline participants’ characteristics that distinguished between trajectories with higher baseline symptoms showing nonresponse versus improving PHQ-9 patterns. Variables included in the analysis consisted of race (identifying as non-Hispanic White and BIPOC), gender (identifying as woman, man, or other), baseline PHQ-9, WHO-5, and GAD-7 scores, and psychosocial flexibility assessed at baseline (ie, Psy-Flex score).

### Ethical Considerations

Participants were recruited prior to joining a group and the discord community. They agreed via the service terms of use and a separate informed consent process to their data being collected for research purposes in a de-identified, aggregated fashion. Research on the feasibility of this support model followed ethical guidelines for human research. Data intended for research use was stripped of identifying information (ie, names, Discord handles, contacts, dates, and account numbers) and were linked across datasets based on anonymized IDs. Regarding data from discord, the server is private (invite-only) and only designated staff and moderators have access to nonanonymized logs. Researchers were only able to access deidentified exports of these. Study analytic procedures were approved as exempt by the NYU School of Medicine Institutional Review Board (i24‑00822). Group members were compensated via free sessions for a month. Participants were free to leave the group at any time.

## Results

### Participants and Demographics

A total of 438 participants that provided follow-up data at their 30-day mark were included in this analysis. Of these, 157 (35.61%) also completed the 60-day survey at exactly 60 days. There was a portion who completed the 60-day survey more than 10 days after the 60-day mark (n=34) or earlier than 20 days before the 60-day mark (n=1). These 35 were dropped from change analyses and demographics. The LGMM analysis was performed with all 438 participants. The demographic and longitudinal change scores were analyzed with n= 403.Of those in the clinically elevated group, 98 (28.91%) completed the 60 day survey. In the subclinical group, 24 (27.90%) completed the 60-day follow-up.

Community demographics reveal an incredibly diverse population with 29.38% (118/403) identifying as BIPOC, 48.64% (196/403) neurodivergent (eg, autism and attention-deficit/hyperactivity disorder), and 25.27% (102/403) noncisgender gender identities. Overall, 45.92% (180/403) of participants identified as women and 28.83% (113/403) identified as men (Table 1). These counts surpass the latest US census demographics ( 82,862,320/331,449,281, 25% BIPOC, 66,289,856/331,449,281, 20% neurodivergent, and 39,773,914/331,449,281 12% gender minorities), highlighting the diversity of video game communities. Demographic information by grouping is reported in Table 2.

**Table 1:** Baseline demographic characteristics of adult gamers (n=403) in a longitudinal online gaming community intervention for loneliness (United States, 2023-2024) compared to 2020 US census population.

Demographic categories^a^			Hero Journey Club participants, (n=403), n (%)		US census 2020 (n=331,449,281), n (%)
**BIPOC^b^**			118 (29.38)		(82,862,320 (25%)
	Indigenous	2 (0.52)		—^c^	
	Asian	14 (3.61)		—	
	Hispanic	22 (5.67)		—	
	Black	15 (3.87)		—	
	Multirace	47 (12.11)		—	
	Others	2 (0.52)		—	
Non-Hispanic White			274 (70.62)		—
Neurodivergent			196 (48.64)		66,289,856 (20)
**Gender identity**			125 (31)		39,773,914 (12)
	Transgender woman	11 (2.81)		—	
	Transgender man	15 (3.83)		—	
	Nonbinary	68 (17.35)		—	
	System	5 (1.28)		—	
	Men	113 (28.83)		165,724,640 (50)	
	Women	180 (45.92)		165,724,640 (50)	

^a^Baseline demographic data collected from 403 adult participants enrolled in the Hero Journey Club online gaming community intervention targeting loneliness. Percentages reflect the diversity of the sample compared to general population estimates from the 2020 US Census.

^b^BIPOC: Black, Indigenous, and People of Color.

^c^Not applicable.

**Table 2:** Longitudinal changes in anxiety (Generalized Anxiety Disorder-7 Scale), depression (Patient Health Questionnaire-9), well-being (World Health Organization-5 Well-Being Index), and psychological flexibility among adult gamers in online gaming community intervention for loneliness at baseline, 30-day, and 60-day follow-up (United States, n=403).

Statistics			Baseline		30 days		60 days
**GAD-7^a^**							
	Mean (SD)	11.23 (5.24)		9.03 (4.99)		8.25 (4.22)	
	Cohen *d*^b^	—^c^		0.43		0.60	
	*t* test (*df*) between time points^d^	—		6.3863 (874)^e^		1.7260 (593)	
**PHQ-9^f^**							
	Mean (SD)	13.37 (6.04)		11.01 (6.15)		10.27 (5.80)	
	Cohen *d*	—		0.39		0.52	
	*t* test (*df*) between time points	—		5.7492 (874)^e^		1.3001 (593)	
**WHO-5^g^**							
	Mean (SD)	7.86 (3.82)		8.22 (4.03)		8.08 (4.44)	
	Cohen *d*	—		–0.092		–0.24	
	*t* test (*df*) between time points	—		–1.3593 (874)		–1.5132 (593)	
**Psy-Flex^h^**							
	Mean (SD)	11.51 (4.30)		11.87 (4.41)		12.55 (4.37)	
	Cohen *d*	—		–0.08		–0.24	
	*t* test (*df*) between time points	—		–1.2327 (874)		–1.6497 (593)	

^a^GAD-7: Generalized Anxiety Disorder-7.

^b^Effect sizes (Cohen *d*) compare baseline scores to each follow-up time point.

^c^Not applicable.

^d^*t* tests compare consecutive time points.

^e^*P*<.001.

^f^PHQ-9: Patient Health Questionnaire-9.

^g^WHO-5: World Health Organization-5 Well-Being Index.

^h^Psy-Flex: Psychological Flexibility Scale.

### Change in Anxiety and Depression

Results for Anxiety and Depression at 60 days are reported in Table 2. At baseline, the average PHQ-9 score was 13.37 (SD 6.04), which falls in the high moderate severity range. At the 60-day follow-up, the average score was low moderate at 10.27 (SD 5.80), with a medium Cohen *d* effect size of 0.52. The average baseline GAD-7 score was 11.23 (SD 5.24), in the low moderate range, and 8.25 (SD 4.22), a high mild, subthreshold score at the 60-day follow-up, with a medium Cohen *d* effect size of 0.60. Psy-Flex and WHO-5 results at 60 days are similarly reported in Table 2. Average baseline scores were 11.51 (SD 4.30) for the Psy-Flex with an average of 12.55 (SD 4.37) at 60 days. The WHO-5 average baseline was 7.86 (SD 3.82) with an average 60-day score of 8.08 (SD 4.44). Cohen *d* for Psy-Flex at 60 days was –0.24 and the WHO-5 Cohen *d* was also –0.24.

**Figure 1:**
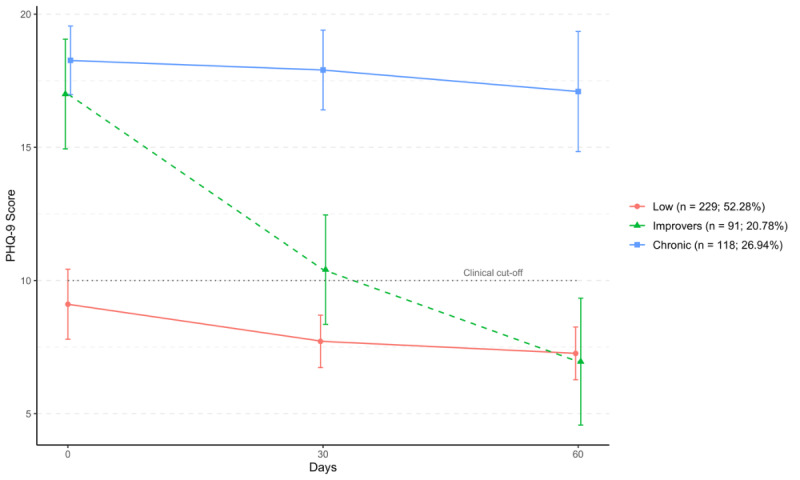
Three heterogeneous trajectories of depressive symptoms (Patient Health Questionnaire-9) over 60 days among adult gamers in online gaming community intervention for loneliness using latent growth mixture modeling (United States, n=438).

Heterogeneous trajectories of depressive symptoms change were estimated using LGMMs. The tree class solution was identified as optimal, with model fit indices reported in the Multimedia Appendices 2-4. Sensitivity analyses suggested no significant differences in likelihood of trajectory membership between survey completer and noncompleters at 30 days (^2^_2_= 0.71 (n=438); *P*=.70) and 60 days (^2^_2_=1.53 (n=438); *P*=.47). The 3 identified trajectories showed distinct patterns of depressive symptom change over 60 days of engagement with the subclinical support program (Figure 1).

The modal trajectory (“Low”; n=229, 52.28%) was characterized by baseline mild to moderate symptoms (baseline PHQ-9 score: mean 8.8, SD 3.67) with a slight decreasing slope over 60 days (60 days PHQ-9 score: mean 7.64, SD 3.87). The second largest heterogeneous subgroup (“Chronic”; n=118, 26.94%) was characterized by baseline moderate to severe symptoms (Baseline PHQ-9 score: mean 18.3, SD 4.03) with no significant change over time (60 days PHQ-9 score: mean 16.78, SD 4.10). The third largest trajectory (“Improvers”; n=91, 20.78%) was characterized by initial baseline moderate to severe PHQ-9 scores (baseline PHQ-9 score: mean 18.52, SD 3.13), which improved to moderate depression levels at 30 days (30 days PHQ-9 score: mean 10.17, SD 4.01) and below the clinical cutoff following 60 days (60 days PHQ-9 score: mean 7.42, SD 4.27).

To identify baseline characteristics that distinguished between participants with moderate to severe baseline depression (n=209) and who exhibited nonresponse versus those who showed symptom improvement, we conducted a logistic regression examining predictors of trajectory membership in the Chronic versus Improvers groups. Results from the analysis indicated that a gender identity other than “woman” was more likely associated with nonimprovement in PHQ-9 scores (odds ratio [OR] 0.42, 95% CI 0.19-0.94; *P*=.04). We further examined potential differences between specific gender identities in post hoc analyses. We conducted multiple pairwise logistic regressions with women as the reference group for each other gender category. Bonferroni correction was applied for 4 pairwise comparisons (α=0.0125). Findings from the post hoc analyses indicated that non-binary individuals (n=68) had significantly lower odds of Improving compared to women (OR 0.25, 95% CI 0.10-0.59; corrected *P*=.009). No significant differences were observed for men, trans women, or trans men compared to women. No other baseline factors significantly distinguished between Chronic and Improvers trajectories. Results are reported in Table 3.

**Table 3:** Baseline predictors of Chronic Elevated Symptoms versus Improvers trajectory membership using logistic regression among adult gamers with moderate-to-severe baseline depression in online gaming community intervention for loneliness (US, n=209).

Variables	Elevated Symptoms Improvers^a^	
	OR^b^ (95% CI)	*P* value
Baseline PHQ-9^c^	1.03 (0.94-1.13)	.49
Baseline GAD-7^d^	0.94 (0.87-1.01)	.10
Baseline WHO-5^e^	0.95 (0.85-1.07)	.40
Baseline PsyFlex^f^	0.93 (0.86-1.00)	.05
Race (non-White)	1.337 (0.70-2.54)	.38
Sex (female)^g^	1.46 (0.72-2.95)	.30
Sex (other)^g^	0.42 (0.19-0.94)	.04

^a^Logistic regression examining baseline demographic and clinical predictors distinguishing participants with persistent elevated depressive symptoms (Chronic trajectory) from those showing symptom improvement (Improvers trajectory) over 60 days. Analysis restricted to participants with moderate-to-severe baseline depression (PHQ-9 ≥10).

^b^OR: odds ratio.

^c^PHQ-9: Patient Health Questionnaire-9.

^d^GAD-7: Generalized Anxiety Disorder-7.

^e^WHO-5: World Health Organization Well-Being Index.

^f^Psy-Flex: Psychological Flexibility Scale.

^g^Reference category: Sex (male).

### Sensitivity Analysis

Sensitivity analyses indicated no significant differences in terms of baseline characteristics between participants who completed all surveys or showed survey nonadherence at 30 and 60 days. Results are reported in the supplementary materials.

## Discussion

### Main Findings

In this longitudinal proof‑of‑concept study, we examined the feasibility and both 30- and 60‑day changes among adults participating in an online gaming therapeutics community. Retention to 60 days was relatively strong for a remote, subclinical program, but this may partly reflect the fact that participants were receiving paid services, which could increase motivation to remain engaged compared with fully free or purely research‑based interventions. Overall, participants showed significant improvements in depressive symptoms, anxiety symptoms, psychological well‑being, and increases in psychological flexibility over 60 days. LGMM identified 3 depressive‑symptom trajectories (Low, Chronic, and Improvers), with an Improvers group showing clinically meaningful reductions in PHQ‑9 scores. The sample was also notably diverse in terms of race, gender, and neurodivergence compared with typical psychotherapy samples, consistent with the program’s aim to reach underserved gamers. Exploratory post hoc analyses further indicated that, among participants with moderate‑to‑severe baseline depression, non‑binary participants were significantly less likely than women to follow the Improvers trajectory, whereas men and binary trans participants did not differ from women. This pattern suggests that, even within a community that is intentionally structured to be inclusive and affirming, some gender minorities may face additional barriers to symptom improvement that require more tailored support.

In this study, depressive and anxiety symptoms were indexed by the PHQ‑9 and GAD‑7, whereas the WHO‑5 captured broader well‑being, including positive mood, vitality, and general life satisfaction. Loneliness functions as a transdiagnostic risk factor that is associated with increased symptoms and poorer social and role functioning across both clinical and nonclinical populations, making it a relevant target for gamers regardless of formal diagnosis. Health‑supporting communities such as the one studied here are hypothesized to reduce loneliness and increase perceived connection, which may in turn support reductions in distress and improvements in subjective well‑being and everyday functioning

Although this intervention took place in an online gaming community, it differs in important ways from the largely unstructured and sometimes toxic gaming spaces described in prior work. The server was intentionally organized as a health‑supporting community, with explicit behavioral guidelines, structured channels and events, and active professional moderation to reduce harassment, discrimination, and other harmful content. These design features likely constrained some of the spontaneity of naturalistic communities but were intended to create conditions in which distressed gamers could safely form relationships and practice new skills.

Loneliness is a rising epidemic in the United States [4,7,8] that has far reaching impacts on physical and mental health [1-6]. Health-supporting communities are designed to alleviate loneliness and the ill health effects associated with loneliness by cultivating individual skill development related to fostering more enduring social connections. These communities are not a replacement for clinical treatment, but work hand-in-hand with existing mental health care, and appear to deliver clinically meaningful results over 60 days. The 30-day and 60-day findings for anxiety, depression, and overall wellness show a positive change in participants' reported functioning.

Importantly, health-supporting communities appear to be acceptable to a diverse set of populations that tend to underuse traditional mental health care. These are all populations that have found it difficult to find meaningful community connection and tend to underuse mental health care [73-77]. In total, 28.83% (113/403) of participants in our program identify as men and 45.92% (18/403) identify as women. Typical demographics rates of mental health care reported ~70% female, ~15% male, and 30% report as BIPOC [78-80]. Demographics beyond these are rarely reported [81]. When compared to typical attendance demographics for psychotherapy, this health-supporting communities for gamers seem to attract a more diverse population. At the same time, our trajectory analyses suggest that some minoritized gender groups, particularly non‑binary participants, may be less likely to experience large symptom reductions over 60 days than women, underscoring that designing “for diverse gamers” requires not only successful recruitment and retention but also ongoing adaptation of content and support structures to address the distinct stressors faced by marginalized identities in gaming spaces.

Video games have historically carried a stigma of harming mental and physical health. Recent research has begun highlighting the potentially health-reinforcing opportunities of video games and their associated communities. This includes physical health as well as for mental health support, such as combating loneliness and the accompanying symptoms of anxiety and depression. Furthermore, some clinicians have demonstrated that using commercial video games as part of treatment can be impactful for clients [32,36]. By incorporating video games as a central part of community and supportive settings, there is potential to assist populations that have often felt uninterested in mental health care services.

LGMM results also reported change over 60 days for 3 distinct groups. While all groups showed improvement over the 60 days, the Improvers group (about 20% 91/438 of the population) showed significant improvement moving from well above clinical cutoff to below clinical cutoff. The only significant difference between the 2 groups that had elevated PHQ scores was gender identity other than male or female. This could be linked to the higher levels of loneliness and depression experienced in these demographics [82].

The post hoc analysis of this LGMM demonstrated that non-binary participants were less likely to be in the Improvers group when compared to women. These results align with current research regarding non-binary individuals reporting poorer mental health conditions when compared to binary individuals (whether trans or cis) [83].

Importantly, the largest group of participants identified by the LGMM reported near threshold depression that was maintained at subthreshold levels for 60 days. The prevention of depression is identified as a longstanding major global concern [84] with psychological and social intervention identified as promising mechanisms of prevention [85]. Social prescribing is likewise a growing area of research for ameliorating and preventing depression episodes [86], though it is still in its infancy and is limited by a lack of robust designs [87]. In person communities and services are often hard to access for some individuals, and a remote option that involves an area of strong interest for some, like video games, could offer an accessible and attractive option for accomplishing the aims of depression prevention.

### Research Positionality and Reflexivity

Members of the research and program team include individuals who identify as gamers, as mental health professionals, and as people with lived experience of mental health challenges. These shared identities likely shaped decisions about program design (eg, emphasis on safety, inclusivity, and subclinical support) and interpretation of findings (eg, attention to both promise and risks of gaming communities). Additionally, the team’s investment in gaming as a potential health‑supporting context may have influenced interpretations of the data.

### Limitations

This data represents a proof-of-concept evaluation of a novel intervention type and is therefore limited in several ways. First, given the longitudinal and real-world nature of this study, survey nonresponse was a primary limitation of the study, with only a reported 30% (157/438) completing the full 60-day observation This limits the ability to fully evaluate longitudinal change. Our LGMM method attempts to estimate for and correct this missingness, but may still over- or underestimate the effects of the intervention.

Future research should include direct measures of loneliness such as the University of California, Los Angeles (UCLA) Loneliness Scale [88,89]. Similarly, our statistical findings were restricted to essential clinical metrics of change over time. Latent structure, clustering and linear models analyses may be warranted in future research with larger samples to better characterize for whom this kind of supportive community is most beneficial. Randomized controlled and decomposition studies would further isolate the relative contribution of program features to promote further program development and efficacy. Future studies should also incorporate systematically collected engagement and platform‑usage data (eg, Discord participation metrics, session attendance, and in‑community behavior markers) that can be securely linked to survey measures to clarify how patterns of engagement relate to symptom and well‑being trajectories over time. There are also opportunities to leverage platform usage data and other rich sources of passive data to understand engagement with the services and community and the impact of that engagement on outcomes that were prevented by privacy concerns related to the platforms being used and limitations on anonymization of platform data, but that could be corrected in future studies. Finally, because participants were enrolled in a paid program and, in some cases, received free sessions as compensation, retention patterns and engagement may differ from what would be observed in unfunded or purely research‑driven implementations, which limits generalizability of the observed retention rates.

### Conclusions

Health‑supporting communities that intentionally combine commercial video games, professionally facilitated groups, and structured online spaces appear to be a promising way to support adults experiencing elevated distress while helping others sustain subthreshold symptom levels. In this proof‑of‑concept study, a substantial subgroup of participants with initially moderate‑to‑severe depressive symptoms followed an ‘Improvers’ trajectory over 60 days, while the largest subgroup maintained mild, subthreshold symptoms, suggesting that such communities may function both as subclinical support and as a potentially preventative context. At the same time, the presence of a Chronic group underscores that this approach will not be sufficient for all participants and highlights the importance of clear positioning as an adjunct or alternative for those who are not ready, able, or willing to engage in traditional psychotherapy.

More broadly, the combination of high levels of racial, gender, and neurodivergent diversity in this sample and the observed changes in symptoms and well‑being suggest that moderated, game‑based communities may help address gaps in reach and relevance for groups that underuse health services. By embedding psychological flexibility processes, social skills practice, and health‑supporting norms within familiar gaming environments and online community structures, this model offers one concrete method of how subclinical supports might complement existing mental health services. Future controlled trials and mechanistic studies will be needed to clarify for whom and under what conditions health‑supporting gaming communities are most beneficial and how they can be integrated into broader prevention and care ecosystems.

## Appendix

Multimedia Appendix 1 STROBE checklist.

Multimedia Appendix 2 A: Moderation rules agreed to by participants of the Discord community.

Multimedia Appendix 3 B: Journey session structure.

Multimedia Appendix 4 Imminent risk protocol.

## Abbreviations

BIPOC: Black, Indigenous, and People of Color
GAD-7: Generalized Anxiety Disorder‑7 Scale
LGBTQIA+: lesbian, gay, bisexual, transgender, queer or questioning, intersex, asexual, and others
LGMM: Latent Growth Mixture Modeling
OR: odds ratio
PHQ-9: Patient Health Questionnaire‑9
Psy-Flex: Psychological Flexibility Scale
STROBE: Strengthening the Reporting of Observational Studies in Epidemiology
UCLA: University of California, Los Angeles
WHO-5: World Health Organization-5 Well‑Being Index
