# Supplementary material for: Gaming‑Based Community Intervention for Loneliness in Adult Gamers: Longitudinal Observational Study
Source: JMIR Form Res. 2026 Feb 10;10:e82428. doi: 10.2196/82428 (PMC12933167; doi:10.2196/82428)
Supplement: Multimedia Appendix 1 [file formative_v10i1e82428_app1.pdf]

STROBE Statement—checklist of items that should be included in reports of observational studies

	Item No.	Recommendation	Page No.	Relevant text from manuscript
**Title and abstract**	1	(*a*) Indicate the study's design with a commonly used term in the title or the abstract	1	The Feasibility of a Gaming Therapeutics Community for Diverse Video Gamers: A longitudinal Observational Study
		(*b*) Provide in the abstract an informative and balanced summary of what was done and what was found	2	The abstract provides an informative summary including background (loneliness as psychological distress), objective (examine feasibility and 30/60-day changes in depression, anxiety, well-being, psychological flexibility), methods (longitudinal observational study, 403-438 participants), results (medium effect sizes for symptom reduction, three trajectory groups identified), and conclusions (feasible intervention, supports further evaluation).
**Introduction**				
Background/rationale	2	Explain the scientific background and rationale for the investigation being reported	4	Loneliness discussed as psychological distress linked to serious medical conditions (diabetes, stroke, heart disease,

				dementia, depression, anxiety, suicide). Organizations like CDC, WHO, APA identify it as critical intervention point. Context provided on gaming communities and their potential for connection despite concerns about toxicity and addiction.
Objectives	3	State specific objectives, including any prespecified hypotheses	2	Examine the feasibility of health-supporting gaming community and characterize 30- and 60-day changes in depression, anxiety, psychological well-being, and psychological flexibility, as well as heterogeneous trajectories of depressive symptoms.
**Methods**				
Study design	4	Present key elements of study design early in the paper	5	Longitudinal observational study design where adults self-enrolled in gaming therapeutics community with assessments at baseline, 30-day, and 60-day timepoints.
Setting	5	Describe the setting, locations, and relevant dates, including periods of recruitment, exposure, follow-up, and data collection		
Participants	6	(*a*) *Cohort study*—Give the eligibility criteria, and the sources and methods of selection of participants. Describe methods of follow-up<br>*Case-control study*—Give the eligibility criteria, and the sources and methods of case ascertainment and control selection. Give the rationale for the choice of cases and controls	5-6	Adults in United States self-enrolled in gaming therapeutics community. Of 438 participants with 30-day data, 403 met inclusion criteria for

		Cross-sectional study—Give the eligibility criteria, and the sources and methods of selection of participants		longitudinal analyses; 157 (35.6%) completed 60-day assessment. Intervention combined professionally facilitated groups, commercial video games, and skills-focused workshops.
		(*b*) *Cohort study*—For matched studies, give matching criteria and number of exposed and unexposed<br>*Case-control study*—For matched studies, give matching criteria and the number of controls per case		
Variables	7	Clearly define all outcomes, exposures, predictors, potential confounders, and effect modifiers. Give diagnostic criteria, if applicable	5-6	Use of validated measures, prospective data collection, systematic assessment procedures.
Data sources/ measurement	8*	For each variable of interest, give sources of data and details of methods of assessment (measurement). Describe comparability of assessment methods if there is more than one group	*5-6*	*438 participants with 30-day data; 403 met inclusion criteria for longitudinal analyses; 157 (35.6%) completed 60-day survey. Sample size determined by enrollment in community intervention program rather than a priori power calculation.*
Bias	9	Describe any efforts to address potential sources of bias	6	Use of validated measures, prospective data collection, systematic assessment procedures.
Study size	10	Explain how the study size was arrived at	5-6	438 participants with 30-day data; 403 met inclusion criteria for longitudinal analyses; 157 (35.6%) completed 60-day survey. Sample size determined by enrollment in community

				intervention program rather than a priori power calculation.

Continued on next page

Quantitative variables	11	Explain how quantitative variables were handled in the analyses. If applicable, describe which groupings were chosen and why	6-76-7	(a) Within-person change scores, Cohen's d, LGMM for trajectory identification. (b) Logistic regression for baseline predictors of trajectory membership; post-hoc analyses for gender comparisons. (c) Missing data: 403 of 438 met inclusion criteria; 157 (35.6%) completed 60-day. (d) Attrition addressed through trajectory modeling. (e) Post-hoc examination of gender differences.
Statistical methods	12	(*a*) Describe all statistical methods, including those used to control for confounding		
		(*b*) Describe any methods used to examine subgroups and interactions		
		(*c*) Explain how missing data were addressed		
		(*d*) *Cohort study*—If applicable, explain how loss to follow-up was addressed<br>*Case-control study*—If applicable, explain how matching of cases and controls was addressed<br>*Cross-sectional study*—If applicable, describe analytical methods taking account of sampling strategy	2, 6-7	Within-person change scores and Cohen's d calculated. LGMM (latent growth mixture modeling) used to identify depressive-symptom trajectories and baseline predictors of non-response versus improvement. Logistic regression used for baseline predictors of trajectory membership; post-hoc analyses for gender comparisons.
		(*e*) Describe any sensitivity analyses		
**Results**				
Participants	13*	(a) Report numbers of individuals at each stage of study—eg numbers potentially eligible, examined for eligibility, confirmed eligible, included in the study, completing follow-up, and analysed	2, 5-6	(a) Demographics (age, gender identity), baseline symptoms (PHQ-9 M=13.37 SD=6.04; GAD-7 M=11.23 SD=5.24;

				WHO-5 M=7.86 SD=3.82; Psy-Flex M=11.51 SD=4.30). (b) 35 excluded for not meeting criteria; 246 lost at 60-day. (c) Follow-up: 30 and 60 days.
		(b) Give reasons for non-participation at each stage		
		(c) Consider use of a flow diagram		
Descriptive data	14*	(a) Give characteristics of study participants (eg demographic, clinical, social) and information on exposures and potential confounders		
		(b) Indicate number of participants with missing data for each variable of interest	2, 5-6	Demographics (age, gender identity), baseline symptoms (PHQ-9, GAD-7, WHO-5, Psy-Flex). 35 excluded for not meeting criteria; 246 lost at 60-day. Follow-up periods: 30 and 60 days.
		(c) *Cohort study*—Summarise follow-up time (eg, average and total amount)		
Outcome data	15*	*Cohort study*—Report numbers of outcome events or summary measures over time	*2-3, 6-72-3, 6-7*	*(a) Cohen's d: PHQ-9 d=0.52, GAD-7 d=0.60, WHO-5 d=0.24, Psy-Flex d=0.24. Logistic regression: gender identity other than woman associated with lower odds of Improvers vs Chronic group (OR=0.42); non-binary vs women (OR=0.25). (b) Outcomes analyzed continuously; trajectories categorical. (c) N/A.*
		Case-control study—Report numbers in each exposure category, or summary measures of exposure		
		Cross-sectional study—Report numbers of outcome events or summary measures		
Main results	16	(*a*) Give unadjusted estimates and, if applicable, confounder-adjusted estimates and their precision (eg, 95% confidence interval). Make clear which confounders were adjusted for and why they were included		

	(*b*) Report category boundaries when continuous variables were categorized	2-3, 6-7	PHQ-9 decreased from M=13.37 (SD=6.04) to M=10.27 (SD=5.80) at 60 days (Cohen's d=0.52). GAD-7 decreased from M=11.23 (SD=5.24) to M=8.25 (SD=4.22) (d=0.60). Psy-Flex increased from M=11.51 (SD=4.30) to M=12.55 (SD=4.37) (d=0.24). LGMM identified three trajectories: Low group (52.3%), Chronic group (26.9%), Improvers group (20.8%). Logistic regression: gender identity other than woman associated with lower odds of Improvers vs Chronic (OR=0.42); non-binary vs women (OR=0.25).
	(*c*) If relevant, consider translating estimates of relative risk into absolute risk for a meaningful time period		

Continued on next page

Other analyses	17	Report other analyses done—eg analyses of subgroups and interactions, and sensitivity analyses	3, 6-73	Observational design without control group, high attrition (64% at 60 days), self-selection, self-report measures, no long-term follow-up. Attrition may favor more engaged/improving participants.
**Discussion**				
Findings support feasibility with medium effect sizes for symptom reduction, but observational design and attrition limit causal conclusions. Results suggest gaming communities may provide scalable support. Further controlled evaluation needed.	18	Summarise key results with reference to study objectives	17	Self-selected US adult gamers in online community may limit generalizability to non-gamers, those without internet access, non-help-seeking individuals, or non-US populations. Diverse gamer sample suggests some generalizability within gaming communities.
Limitations	19	Discuss limitations of the study, taking into account sources of potential bias or imprecision. Discuss both direction and magnitude of any potential bias	21	Self-selected US adult gamers in online community may limit

				generalizability to non-gamers, those without internet access, non-help-seeking individuals, or non-US populations. Diverse gamer sample suggests some generalizability within gaming communities.
Interpretation	20	Give a cautious overall interpretation of results considering objectives, limitations, multiplicity of analyses, results from similar studies, and other relevant evidence	17-21	Findings support feasibility with medium effect sizes for symptom reduction, but observational design and attrition limit causal conclusions. Results suggest gaming communities may provide scalable support. Further controlled evaluation needed.
Generalisabilit y	21	Discuss the generalisability (external validity) of the study results	17-21	Self-selected US adult gamers in online community may limit generalizability to non-gamers, those without internet access, non-help-seeking individuals, or non-US populations. Diverse gamer sample suggests some generalizability within gaming communities.
**Other information**				
Funding	22	Give the source of funding and the role of the funders for the present study and, if applicable, for the original study on which the present article is based	24	Funding sources and role of funders would be detailed in full manuscript acknowledgments/funding section.

*Give information separately for cases and controls in case-control studies and, if applicable, for exposed and unexposed groups in cohort and cross-sectional studies.

Note: An Explanation and Elaboration article discusses each checklist item and gives methodological background and published examples of transparent reporting. The STROBE checklist is best used in conjunction with this article (freely available on the Web sites of PLoS Medicine at http://www.plosmedicine.org/, Annals of Internal Medicine at http://www.annals.org/, and Epidemiology at http://www.epidem.com/). Information on the STROBE Initiative is available at www.strobe-statement.org.
