# Supplementary material for: Gaming‑Based Community Intervention for Loneliness in Adult Gamers: Longitudinal Observational Study
Source: JMIR Form Res. 2026 Feb 10;10:e82428. doi: 10.2196/82428 (PMC12933167; doi:10.2196/82428)
Supplement: Multimedia Appendix 2 [file formative_v10i1e82428_app2.docx]

**Appendix A**

*Moderation Rules Agreed to by Participants of the Discord Community*

1. Commitment to Safety: We prioritize your safety above all. Stay vigilant for any signs of inappropriate behavior, such as secretive messaging or uncomfortable questions. If you feel uneasy, please block the individual, report the situation to us, and let our support team guide you through the next steps. We enforce a strict zero-tolerance policy towards grooming and will take decisive actions, including reporting to authorities to ensure our community remains a safe space for everyone.
2. Guidelines for Discussions: Our community thrives on respect and inclusivity, which means certain topics are best left outside our conversations for everyone's comfort:
   1. Politics and Religion
   2. Substance Misuse (with the exception of designated support sessions),
   3. Sharing Personal Information,
   4. Hate Speech and Threats,
   5. Sexual Topics,
3. Even as an 18+ server, we encourage polite language and respect towards all members.
4. Positive Interactions: We believe in fostering a supportive and compassionate environment. Any form of aggression or disrespect is contrary to our values and won't be tolerated.
5. Communication Etiquette: We appreciate messages that are thoughtful and contribute constructively to our discussions. Let's keep our exchanges meaningful.
6. Building Friendships: Our focus is on nurturing support and friendships within our community. We kindly ask that you refrain from seeking romantic relationships here.
7. Respecting Privacy: Please protect the privacy and safety of minors by not sharing their images in our community.
8. Inclusive Conversations: To ensure everyone can participate and understand, please keep all conversations in English and appropriate for a diverse group.
9. Following Leadership: Our staff, leaders, and moderators are here to ensure your experience is positive. Please respect their guidance and understand they'll respond as promptly as possible. Repeated pinging is discouraged.
10. External Links and DMs: To maintain the safety and integrity of our community, we prohibit invitations to external servers and ask that you keep direct messaging turned off. This helps all conversations remain transparent and within our community space.
