# Supplementary material for: Gaming‑Based Community Intervention for Loneliness in Adult Gamers: Longitudinal Observational Study
Source: JMIR Form Res. 2026 Feb 10;10:e82428. doi: 10.2196/82428 (PMC12933167; doi:10.2196/82428)
Supplement: Multimedia Appendix 3 [file formative_v10i1e82428_app3.docx]

**Appendix B**

*Journey Session Structure*

There are three to five people placed in each journey group. Members are matched into groups based on their reported goals, games played, gender, sex, facilitator preferences, age, schedule availability, and other factors. If only three participants show up for a session, guides have the option of either continuing if the other participants are ok with a smaller group, or cancelling the session and participants are able to submit a ticket for a refund.

Journey sessions are 80 minutes long. And scheduled with a 10 minute break after those 80 minutes. In the first session, the journey guide leads participants through introductions, asking them a series of questions including, preferred name and pronouns, area of interest to work on, fun fact or gaming interests, other hobbies and interests, and one thing each participant wished everyone knew about mental health. Guides then describe the group agreements which include:

- Speaking from the “I” experience
- Listen to understand, and challenge the idea, not the person.
- Ask consent and use trigger warnings
- Flow in and flow out.
- Practice inviting people to share if you share a lot or challenge yourself to share if you typically don’t share that much.

After each participant agreed to the group rules, they collectively made the decision to either game in the session or not. Groups also got set up in the game of choice at that time. After this initial setup, all journey sessions followed the same structure: 1. Check-in where participants were asked to share the best part of the week, the worst part of the week, and something they’re looking forward to. Journeyers could also share how they are feeling on a scale of 1-10 and were asked to provide reasoning for the number they picked. 2. After the check-in, guides identified a theme from the check-in and asked if the group wanted to explore this theme. They could also ask the journeyers what they needed from the group that day. 3. At the end of the session, guides provide takeaways if needed. Journey sessions met weekly.
