# Supplementary material for: Gaming‑Based Community Intervention for Loneliness in Adult Gamers: Longitudinal Observational Study
Source: JMIR Form Res. 2026 Feb 10;10:e82428. doi: 10.2196/82428 (PMC12933167; doi:10.2196/82428)
Supplement: Multimedia Appendix 4 [file formative_v10i1e82428_app4.docx]

**Appendix C**

*Imminent Risk Protocol*

Within session, guides were instructed to not ask about suicidal ideation (SI). However, if participants volunteered this information, guides were instructed to not pause the session or stop the participant from discussing it further. Guides were also instructed to not perform a risk assessment or safety plan with the participant, because they are not acting as treating clinicians in this setting and instead follow a standardized referral and escalation protocol. Rather, guides were instructed to direct participants to the crisis-resource channel and to alert the director of clinical operations. If SI was active and imminent, guides were instructed to have participants stop the call and immediately call 911. The hierarchy of action is as follows:

1. Ask the journeyer to disconnect from the session and call 911
2. Check the journeyer’s state of residence
3. If the journeyer is in a duty-to-warn state, do an online search for the state’s duty-to-warn number. If unable to be found, use PSAP to call for a welfare check.
4. Encourage the journeyer to utilize the crisis-resources channel
5. If they are unable or unwilling to call, guides were instructed to call themselves on their behalf
6. Alert the Director of Clinical Operations
7. Document all actions in group session notes

If threats are externally focused (homicidal thoughts/behaviors), guides are mandated to call 911 and ask the journeyer to call 911. Guides were also asked to pause the session, letting other journeyers know that the guide is stepping away to make sure the journeyer in question is getting the support they need.
